# Myocardial ultrasonic tissue characterization in patients with thyroid dysfunction

**DOI:** 10.1186/1476-7120-8-15

**Published:** 2010-04-23

**Authors:** Minna MD Romano, Léa MZ Maciel, Oswaldo C Almeida-Filho, Antonio Pazin-Filho, André Schmidt, Benedito C Maciel

**Affiliations:** 1 Division of Cardiology, Department of Internal Medicine, University Hospital, Medical School of Ribeirão Preto, University of São Paulo, Brazil; 2 Division of Endocrinology, Department of Internal Medicine, University Hospital, Medical School of Ribeirão Preto, University of São Paulo, Brazil; 3 Division of Clinical Emergency, Department of Internal Medicine, University Hospital, Medical School of Ribeirão Preto, University of São Paulo, Brazil

## Abstract

**Background:**

Structural myocardial abnormalities have been extensively documented in hypothyroidism. Experimental studies in animal models have also shown involvement of thyroid hormones in gene expression of myocardial collagen. This study was planned to investigate the ability of ultrasonic tissue characterization, as evaluated by integrated backscatter (IBS), to early identify myocardial involvement in thyroid dysfunction.

**Patients and Methods:**

We studied 15 patients with hyperthyroidism (HYPER), 8 patients with hypothyroidism (HYPO), 14 patients with subclinical hypothyroidism (SCH) and 19 normal (N) subjects, who had normal LV systolic function. After treatment, 10 HYPER, 6 HYPO, and 8 SCH patients were reevaluated. IBS images were obtained and analyzed in parasternal short axis (papillary muscle level) view, at left ventricular (LV) posterior wall. The following IBS variables were analyzed: 1) the corrected coefficient (CC) of IBS, obtained by dividing IBS intensity by IBS intensity measured in a rubber phantom, using the same equipment adjustments, at the same depth; 2) cardiac cyclic variation (CV) of IBS - peak-to-peak difference between maximal and minimal values of IBS during cardiac cycle; 3) cardiac cyclic variation index (CVI) of IBS - percentual relationship between the cyclic variation (CV) and the mean value of IBS intensity.

**Results:**

CC of IBS was significantly larger (p < 0.05) in HYPER (1.57 ± 0.6) and HYPO (1.53 ± 0.3) as compared to SCH (1.32 ± 0.3) or N (1.15 ± 0.27). The CV (dB) (HYPO: 7.5 ± 2.4; SCH: 8.2 ± 3.1; HYPER: 8.2 ± 2.0) and the CVI (HYPO: 35.6 ± 19.7%; SCH: 34.7 ± 17.5%; HYPER: 37.8 ± 11.6%) were not significantly different in patients with thyroid dysfunction as compared to N (7.0 ± 2.0 and 44.5 ± 15.1%).

**Conclusions:**

CC of IBS was able to differentiate cardiac involvement in patients with overt HYPO and HYPER who had normal LV systolic function. These early myocardial structural abnormalities were partially reversed by drug therapy in HYPER group. On the other hand, although mean IBS intensity tended to be slightly larger in patients with SCH as compared to N, this difference was not statistical significant.

## Introduction

Information regarding myocardial ultra-structure and composition can be provided by ultrasonic tissue characterization, a non-invasive technique which is capable of detecting and quantifying acoustic properties of myocardial tissue [1]. The intensity of the IBS is related to physical properties of myocardium and is influenced by tissue components (collagen, water, fat) and spatial distribution of these components [1,2]. In addition, intensity of the IBS also depends on ultra-sound system settings and on ultrasound attenuation [3,4]. A number of investigations have shown that morphologic changes in myocardium occurring in various diseases are associated with increased integrated backscatter (IBS) [5-10]. During cardiac contraction and relaxation, a cardiac cycle-dependent variation of myocardial IBS can be documented. The magnitude of cyclic variation of IBS is an additional acoustic parameter, which is more strictly correlated with myocardial contractile function [11,12] and independent of ultrasound system settings.

It has been consensually recognized that thyroid dysfunction is associated to remarkable changes in cardiac structure and function [13,14]. Thyroid hormones effects on the heart are exerted both by direct influence at cellular level, involving nuclear and extranuclear mechanisms, and by indirect interactions with the renin-angiotensin-aldosterone system, sympathetic nervous system, natriuretic peptides and erythropoietin secretion [13,14]. There is experimental evidence showing a regulatory involvement of thyroid hormones on interstitial protein gene expression, modulating production of collagen type I, a main component of the heart matrix, which plays an important role in maintaining the integrity of myocardial function [15]. Despite of that, there are very few studies [16-19] noninvasively analyzing the ultrastructural characteristics of heart muscle using ultrasound. Therefore, this study was designed to investigate the ability of ultrasonic tissue characterization to identify early myocardium involvement in patients with thyroid dysfunction, including overt hypothyroidism and hyperthyroidism, as well as subclinical hypothyroidism, before and after drug therapy.

## Methods

### Study group

Fifty-six subjects, of both sexes, were evaluated in this study, including 19 healthy volunteers (16 male), mean age 43 ± 8.4 years (mean ± SD) (**Controls**) and 37 patients with thyroid dysfunction divided in the following groups: **Hyperthyroidism **(**HYPER**), including 15 female patients, mean age 30.3 ± 7.9 years; **Hypothyroidism **(**HYPO**), consisting of 8 patients (5 female), mean age 40 ± 7.8 years, and **Subclinical Hypothyroidism **(**SCH**), comprising 14 female patients, mean age 41.2 ± 8.0 years. Clinical diagnosis of newly untreated thyroid dysfunction was confirmed by immunoassay determination of hormone levels (TSH, FT_4 _and FT_3_). None of these patients had any clinical, electrocardiographic or echocardiographic evidence of cardiovascular disease. At least six months after clinical and metabolic control by drug therapy, 10 HYPER, 6 HYPO, and 8 SCH patients were reevaluated. Laboratory reference values for thyroid hormones were: TSH - 0.5 to 4.5 μU/ml; FT_4 _- 07 to 1.7 ng/dl and FT_3 _- 1.5 to 4.1 pg/ml. Patients presenting overt and subclinical hypothyroidism were treated with graded substitutive doses of levothyroxine, while patients with overt hyperthyroidism received methimazole. The protocol was approved by the institutional ethics committee. All subjects who were included in this investigation provided written informed consent.

### Protocol

M-mode and 2D echocardiograms and Doppler analysis were performed in all subjects using a commercially available ultrasound system (Philips Sonos 5500; Andover, Massachussets) with a S4 (2-4 MHz) transducer. Two dimensional images were obtained in parasternal long-axis and short axis views and in apical four and two chamber views. M-mode measurements of aorta, left atrium, right ventricle, left ventricle diastolic and systolic dimensions in addition to diastolic dimension of the interventricular septum and the left ventricular posterior wall were obtained in all subjects. Global left ventricular systolic function was evaluated by quantifying ejection fraction using Simpson's method on apical two and four chamber views.

**Figure 1:**
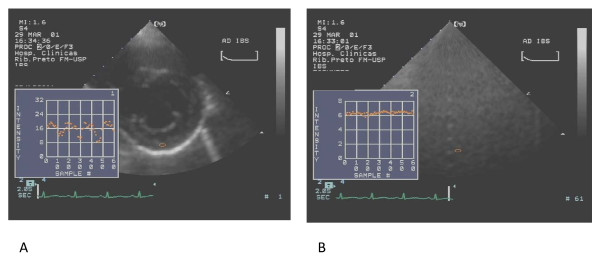
**IBS curves from posterior wall of the left ventricle (A) and from a rubber phantom, maintaining sample (arrows) in the same depth and same system settings (B)**.

Two dimensional ultrasonic backscatter images of left ventricle were obtained using an on-line acoustic densitometry package incorporated in the same imaging system from paraesternal short axis view at papillary muscle level. This ultrasound system provided two-dimensional echocardiographic images in which the gray level is represented proportional to the integrated backscatter. Images were captured on a cine loop (2 seconds at 30 frames/sec), displayed and stored on optical disk. Backscatter was measured in decibels, ranging from 10 to 40 dB, from an elliptical region of interest, 11 × 11 to 31 × 31 pixels. The largest possible region of interest was placed at the center of the posterior wall myocardial segment, taking care to avoid bright specular echoes from endocardium or epicardium and to keep the region of interest within the myocardial borders during cardiac cycle. For each view evaluated, a cine-loop from a rubber phantom, using the same system settings, was also acquired (Figure 1). Each segment was evaluated in triplicate and the mean values of each variable were used in the statistical analysis.

For each myocardial segment, the acoustic densitometry package provided the following variables: a mean integrated backscatter and the magnitude of cyclic variation of IBS. Integrated backscatter was calibrated for system settings and tissue attenuation by dividing mean intensity of IBS, at posterior left ventricular segment, by IBS mean intensity measured in the rubber phantom image, acquired using the same system settings at the same depth as the LV segment. This variable was the corrected coefficient (CC) of IBS. Magnitude of cyclic variation (CV) of IBS was obtained as the difference between the average peak and average nadir values of IBS. We also calculated the cardiac cyclic variation index (CVI) of IBS, as measured by the percentual relationship between CV and the mean value of IBS intensity.

### Statistical analysis

All data are presented as mean ± standard deviation. Non-parametric Kruskal-Wallis test with Dunn's multiple comparisons test, when applicable, was used to compare continuous variables. Wilcoxon non-parametric test was used to compare patients with thyroid dysfunction before and after treatment. Simple linear regression analysis was used for evaluation of relationship between hormone levels and IBS variables. A p value < 0.05 was considered as statistically significant.

## Results

Clinical and hormonal data of patients with thyroid dysfunction and controls are shown in Table 1. Subjects with hyperthyroidism presented a significantly lower (p < 0.01) mean age (30.3 ± 7.9 years; mean ± SD) as compared to control group (43 ± 8.4 years), patients with overt (40.0 ± 7.8 years) or subclinical hypothyroidism (41.2 ± 8.0 years). Predominance of female patients was observed in thyroid dysfunction groups while control group was composed by a majority of male subjects. By selection, patients presenting hyperthyroidism had suppressed TSH levels and increase FT_4 _and FT_3 _values while patients with overt hypothyroidism showed very high levels of TSH and reduced values of FT_4_; on the other hand, a slight increase of TSH and FT_4 _levels in the normal reference range were observed in patients with subclinical hypothyroidism.

**Table 1:** Clinical, hormonal and echocardiographic variables (mean ± SD) in patients with thyroid dysfunction and controls

	Hyperthyroidism (n = 15)	Hypothyroidism (n = 8)	Subclinical Hypothyroidism (n = 14)	Controls (n = 19)
**Age (yrs)**	30.3 ± 7.9^a^	40 ± 7.8	41.2 ± 8.0	43 ± 8.4
**Sex (F:M)**	15:0	5:3	14:0	3:16
**BSA (m^2^)**	1.6 ± 0.2	1.8 ± 0.2	1.6 ± 0.1	1.8 ± 0.1
**HR (beats/min)**	90.6 ± 19.3	66.3 ± 10.5	77.5 ± 13.1	72.8 ± 15.4
**EF (%)**	70.2 ± 5.4	69.4 ± 5.4	70.1 ± 6.6	66.2 ± 7.3
**LVMI (g/m^2^)**	76.5 ± 14.7	85.9 ± 17.0	67.8 ± 9.0	89.8 ± 20.2
**LVd (cm)**	4.9 ± 0.2	4.8 ± 0.3	4.7 ± 0.3	4.9 ± 4.6
**TSH (mU/L)**	0.01 ± 0.009	156.5 ± 97.7	12.6 ± 7.8	**-**
**FT_4_(ng/dl)**	4.24 ± 1.6	0.2 ± 0.07	0.9 ± 0.2	**-**
**FT_3_(pg/ml)**	11.5 ± 8.0	-	-	-

yrs = years; F = female; M = male; BSA = body surface area; HR = heart rate; EF = ejection fraction; LVd = left ventricular diastolic dimension; LVMI = left ventricular mass index; LVd = left ventricle diastolic dimension; TSH = thyroid stimulator hormone; FT_4 _= free T_4_; FT_3 _= free T_3_; ^a^:p < 0.01 vs hypothyroidism, subclinical hypothyroidism and controls.

On baseline Doppler echocardiography study (Table 1), left ventricular diastolic dimension, left ventricular mass index and ejection fraction had comparable values for normal subjects and patients with thyroid dysfunction.

**Figure 2:**
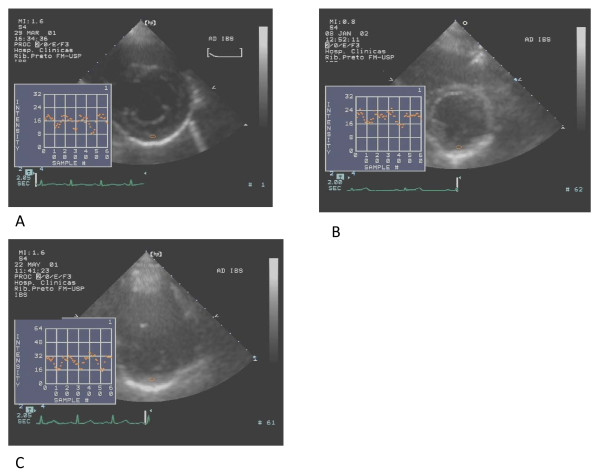
**IBS curves of patients with subclinical hypothyroidism (A); hypothyroidism (B) and hyperthyroidism (C)**.

**Figure 3:**
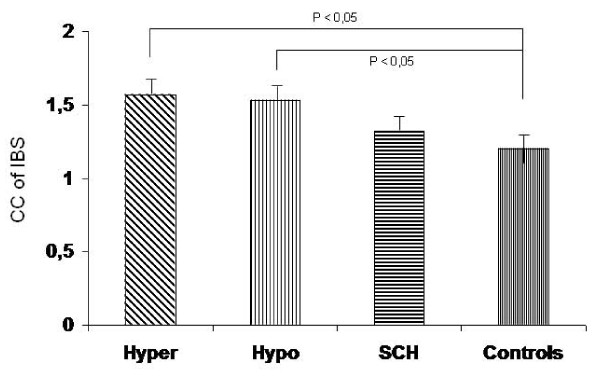
**Integrated backscatter (IBS) variables (mean ± SD) in patients with thyroid dysfunction and controls**. CC of IBS = corrected coefficient of IBS; Hyper = hyperthyroidism; Hypo = hypothyroidism; SCH = subclinical hypothyroidism.

The corrected coefficient of IBS was significantly higher among patients with overt hyperthyroidism and hypothyroidism (Figure 2) as compared to normal subjects. Even though there was a clear tendency for higher values of this myocardial texture index in patients with subclinical hypothyroidism, statistical significance could not be demonstrated (Table 2, Figure 3).

**Table 2:** Integrated backscatter (IBS) variables (mean ± SD) in patients with Thyroid Dysfunction and Controls

	Hyperthyroidism (n = 15)	Hypothyroidism (n = 8)	Subclinical Hypothyroidism (n = 14)	Controls (n = 19)
CC of IBS	1.57 ± 0.6 ^a^	1.53 ± 0.3 ^a^	1.32 ± 0.3	1.2 ± 0.3
CV (dB)	8.2 ± 2.0	7.5 ± 2.4	8.2 ± 3.1	7 ± 2.0
CVI (%)	37.8 ± 11.6	35.6 ± 19.7	34.7 ± 17.5	44.5 ± 15.1

^a ^= p < 0.05 vs controls; CC of IBS = correct coefficient of IBS; CV = absolute cyclic variation of IBS; CVI = percentual relationship between CV and mean value of IBS

When magnitude of cyclic variation (CV) of the integrated backscatter and cardiac cyclic variation index (CVI) of IBS were compared in normal subjects and different groups of thyroid dysfunction patients, these IBS indices were not consistently different between groups (Table 2).

Considering that the corrected coefficient of IBS in the control group have shown a normal distribution, we compared individual values for this variable in the thyroid dysfunction patients in the range of mean ± 2 SD. It was observed that 4 out 15 patients with hyperthyroidism (27%) and 1 out 14 patients (7.1%) of patients with subclinical hypothyroidism had corrected coefficients of IBS higher than the upper normal limit while almost all patients with overt hypothyroidism had IBS values close to this upper limit.

Table 3 shows clinical, echocardiographic and hormonal data for thyroid dysfunction patients who were evaluate before and after clinical treatment. Besides significant changes after treatment showing return of thyroid hormone levels within normal range, a significant reduction of left ventricular diastolic dimension was documented after clinical control of disease.

**Table 3:** Clinical, Hormonal and Echocardiographic variables (mean ± SD) in patients with Thyroid Dysfunction before and after treatment

	Hyperthyroidism (n = 10)		Hypothyroidism (n = 6)		Subclinical Hypothyroidism (n = 8)	
	**Baseline**	**Treatment**	**Baseline**	**Treatment**	**Baseline**	**Treatment**
HR (min^-1^)	90.7 ± 23.5	80 ± 16.4	67.3 ± 11.1	69.7 ± 13.8	78.8 ± 14.3	78.3 ± 10.7
EF (%)	70.4 ± 5.6	71.7 ± 5.1	70.7 ± 4.7	71.0 ± 5.2	71.2 ± 5.4	72 ± 4.4
LVMI (g/m^2^)	72.2 ± 11.1	68.0 ± 11.5	82.0 ± 15.7	80.7 ± 16.0	70.8 ± 9.7	69.2 ± 10.2
LVd (cm)	4.9 ± 0.3	4.7 ± 0.3 ^a^	4.9 ± 0.2	4.9 ± 0.2	4.8 ± 0.3	4.8 ± 0.3
TSH (mU/L)	0.009 ± 0.01	2.1 ± 1.4 ^b^	141.2 ± 96.9	1.7 ± 1.5 ^a^	10.6 ± 7.1	2.4 ± 1.3^a^
FT_4 _(ng/dl)	4.3 ± 1.6	1.2 ± 0.2 ^b^	0.2 ± 0.08	1.53 ± 0.2^a^	1.0 ± 0.2	1.4 ± 0.3 ^a^
FT_3 _(pg/ml)	13.5 ± 9.2	3.1 ± 0.9	-	-	-	-

^a ^= p < 0.05; ^b ^= p < 0.005 vs baseline

HR = heart rate; EF = ejection fraction; LVMI = left ventricular mass index; LVd = left ventricular diastolic dimension; TSH = thyroid stimulator hormone; FT_4 _= free T_4_; FT_3 _= free T_3_

Mean values of the corrected coefficient of IBS tended to reduce in patients with overt hyperthyroidism and hypothyroidism after treatment, although this reduction was not statistically significant. Despite a clear tendency of increasing values of the absolute MCV of the integrated backscatter and cardiac cyclic variation index (CVI) of IBS after drug therapy were documented in all groups of thyroid dysfunction, statistical significance (p < 0.05) was reached only by the magnitude of cyclic variation of IBS for patients with overt hypothyroidism (Table 4). In addition, most of those patients who were evaluated after drug therapy presented a marked reduction of the corrected coefficient of IBS.

**Table 4:** Integrated backscatter (IBS) variables (mean ± SD) in patients with Thyroid Dysfunction before and after treatment

	Hyperthyroidism (n = 10)		Hypothyroidism (n = 6)		Subclinical Hypothyroidism (n = 8)	
	**Baseline**	**Treatment**	**Baseline**	**Treatment**	**Baseline**	**Treatment**
CC of IBS	1.7 ± 0.7	1.3 ± 0.2 ^b^	1.6 ± 0.2	1.4 ± 0.3	1.4 ± 0.2	1.4 ± 0.2
CV (dB)	8.1 ± 2.1	11.1 ± 4.7	8.3 ± 2.0	12.3 ± 2.9 ^a^	8.2 ± 2.7	10.9 ± 4.3
CVI (%)	39.1 ± 11.5	41.5 ± 14.2	41.2 ± 19.6	51.8 ± 16.2	36.1 ± 20.4	52.3 ± 18.4

^a ^= p < 0.05; ^b ^= p < 0.08 vs baseline; CC of IBS = correct coefficient of IBS; CV = absolute cyclic variation of IBS; CVI = percentual relationship between CV and mean value of IBS

## Discussion

The results of the present investigation have shown that calibrated myocardial integrated backscatter, as measured by the corrected coefficient of IBS, was able to early demonstrate myocardial texture abnormality in patients with overt hyperthyroidism and hypothyroidism who had normal left ventricular systolic function, but not in patients with subclinical hypothyroidism. In addition, myocardial cyclic variation of IBS and cyclic variation index were not able to differentiate thyroid dysfunction patients from normal subjects. On the other hand, clinical and metabolic control of thyroid dysfunction tended to reduce IBS intensity and to increase cyclic variation of IBS in hyperthyroid and hypothyroid patients.

The increased myocardial echoreflectivity we documented in patients with overt hypothyroidism has been previously reported [17]. Taking into account the recognized correlation between ultrasound backscatter intensity and histologically quantified myocardial fibrosis [20], this finding is consistent with experimental data suggesting involvement of thyroid hormones in regulating myocardial fibrosis by modulating collagen synthesis [15].

On the other hand, to the best of our knowledge, the increased myocardial texture observed in patients presenting overt hyperthyroidism has not been previously described. A previous investigation [17] comparing patients with hyperthyroidism to normal subjects did not find significant changes in cardiac echoreflectivity. Di Bello et al [21], have already demonstrated an increase in CV of IBS in subclinical hyperthyroidism without any differences in IBS intensity versus control group. In contrast, our data suggest that at least some patients with hyperthyroidism have a degree of myocardial fibrosis, reflected by increased CC of IBS, which tended to reverse after treatment. Considering that: 1) myocardial fibrosis depends on a complex relationship between stimulatory (including: angiotensin II, endothelin I, catecholamines and aldosterone) and inhibitory factors acting on the collagen production [22]; 2) renin-angiotensin-aldosterone system is activacted in patients with hyperthyroidism [13,14]; 3) thyroid hormones may influence the responsiveness and sensitivity of cardiac tissue to normal sympathetic stimulation [13]; it is reasonable to admit that these mechanisms could mediate the increased myocardial texture observed in these patients.

Our results regarding myocardial cyclic variation of IBS and cyclic variation index are in disagreement with previous publications [16,18,19,21] which have shown significant lower cyclic variation in patients with subclinical hypothyroidism while our data were not able to differentiate thyroid dysfunction patients from normal subjects. Those observations have been considered to indicate an early myocardial dysfunction in patients presenting subclinical hypothyroidism.

To conciliate our results with previous publications, we should consider a number of factors. Certainly, a heterogeneous composition of the patient population, depending on different disease duration, baseline hormone levels, clinical stability and severity of dysfunction, could contribute to the conflicting results. In addition, the capability of ultrasonic tissue characterization to detect early and minor ultra structural changes in myocardial diseases has not been completely evaluated. In part, this lack of sensitivity is probably related to the large variability of individual values of this variable, documented even in normal subjects, as previously published [9]. There are also differences regarding methodology for evaluating ultrasound tissue characterization. The use of absolute integrated backscatter to evaluate the acoustic properties of the myocardium in different clinical conditions has limited value due to influence of ultrasound system settings and tissue attenuation [3,4] on quantification of IBS variables. To overcome this limitation, several methods have been employed to calibrate the absolute value of IBS by a reference value, derived from ventricular cavity [6], pericardium [7,8,23,24] or from a rubber phantom [25], as we utilized in this investigation. Although absolute IBS values derived from ventricular cavity or pericardium could be adequate for reducing the influence of the ultrasound system settings, they are limited to correct the effects of ultrasound attenuation.

Despite the marked predominance of female patients in thyroid dysfunction groups as opposed to preponderance of male subjects in the control group, it should be mentioned that there are not any data showing gender influence on myocardial tissue characterization variables.

Regression of ultrasonic myocardial texture alterations have been already documented in clinical scenarios of hypertrophy regression [26,27]. Although our results showed a trend toward regression of both IBS indices (CC IBS and CV) after treatment of patients with overt hypothyroidism or hyperthyroidism, this was only statistical significant in CV of hypothyroidism patients. This lack of statistical significance may be related to the limited numbers of patients evaluated after treatment.

Ultrasonic backscatter can provide important information regarding structural and functional alterations in the myocardium of patients with different types of heart disease. In this investigation, this technique was able to document early myocardial structural abnormalities in patients with overt hyperthyroidism or hypothyroidism, and CCIBS was partially reversed by drug therapy in patients with hyperthyroidism.

## Competing interests

The authors declare that they have no competing interests.

## Authors' contributions

MMDR quantified the IBS images, participated in the sequence alignment and drafted the manuscript; LMZM participated in the sequence alignment and selection of patients; OCF collected all echocardiographic data and participated in the design of the study; APF participated in the design of the study and reviewed the final text; AS participated in the design of the study, performed the statistical analysis and reviewed the final text; BCM conceived of the study and participated in its design and coordination. All authors read and approved the final manuscript.
